# Retraction of “MICAL2 Mediates p53 Ubiquitin Degradation through Oxidating p53 Methionine 40 and 160 and Promotes Colorectal Cancer Malignance”

**DOI:** 10.7150/thno.55371

**Published:** 2021-01-01

**Authors:** Jinping Lu, Yuejin Li, Yuanzhong Wu, Shan Zhou, Chaojun Duan, Zigang Dong, Tiebang Kang, Faqing Tang

**Affiliations:** 1 Department of Clinical Laboratory, Hunan Cancer Hospital & the Affiliated Cancer Hospital of Xiangya School of Medicine, Central South University, 410013, Changsha, China; 2 State Key Laboratory of Oncology in South China and Department of Experimental Research, Sun Yat-sen University Cancer Center, Guangzhou 510060, Guangdong, China; 3 Department of Clinical Laboratory and Medical Research Center, Zhuhai Hospital, Jinan University, Zhuhai 519000, Guangdong, China; 4 Hormel Institute, University of Minnesota, 801 16th Avenue NE, Austin, MN 55912

The Editor-In-Chief of Theranostics, in consultation and agreement with the editorial board members, retracts the article “MICAL2 Mediates p53 Ubiquitin Degradation through Oxidating p53 Methionine 40 and 160 and Promotes Colorectal Cancer Malignance” 1 on the basis of questions related to several figures. The concerns about the figures also raise questions about the conclusions within the paper.
